# Transcriptomic and physiological analysis of *atractylodes chinensis* in response to drought stress reveals the putative genes related to sesquiterpenoid biosynthesis

**DOI:** 10.1186/s12870-024-04780-8

**Published:** 2024-02-06

**Authors:** Shanshan Ma, Chengzhen Sun, Wennan Su, Wenjun Zhao, Sai Zhang, Shuyue Su, Boyan Xie, Lijing Kong, Jinshuang Zheng

**Affiliations:** Hebei Key Laboratory of Crop Stress Biology, Hebei Normal University of Science & Technology, Qinhuangdao, Hebei 066004 China

**Keywords:** *Atractylodes chinensis* (DC) Koidz., Drought stress, Transcriptome, Differentially expressed genes, Sesquiterpenoid

## Abstract

**Background:**

*Atractylodes chinensis* (DC) Koidz., a dicotyledonous and hypogeal germination species, is an important medicinal plant because its rhizome is enriched in sesquiterpenes. The development and production of *A. chinensis* are negatively affected by drought stress, especially at the seedling stage. Understanding the molecular mechanism of *A. chinensis* drought stress response plays an important role in ensuring medicinal plant production and quality. In this study, *A. chinensis* seedlings were subjected to drought stress treatment for 0 (control), 3 (D3), and 9 days (D9). For the control, the sample was watered every two days and collected on the second morning after watering. The integration of physiological and transcriptomic analyses was carried out to investigate the effects of drought stress on *A. chinensis* seedlings and to reveal the molecular mechanism of its drought stress response.

**Results:**

The malondialdehyde, proline, soluble sugar, and crude protein contents and antioxidative enzyme (superoxide dismutase, peroxidase, and catalase) activity were significantly increased under drought stress compared with the control. Transcriptomic analysis indicated a total of 215,665 unigenes with an average length of 759.09 bp and an N50 of 1140 bp. A total of 29,449 differentially expressed genes (DEGs) were detected between the control and D3, and 14,538 DEGs were detected between the control and D9. Under drought stress, terpenoid backbone biosynthesis had the highest number of unigenes in the metabolism of terpenoids and polyketides. To identify candidate genes involved in the sesquiterpenoid and triterpenoid biosynthetic pathways, we observed 22 unigene-encoding enzymes in the terpenoid backbone biosynthetic pathway and 15 unigene-encoding enzymes in the sesquiterpenoid and triterpenoid biosynthetic pathways under drought stress.

**Conclusion:**

Our study provides transcriptome profiles and candidate genes involved in sesquiterpenoid and triterpenoid biosynthesis in *A. chinensis* in response to drought stress. Our results improve our understanding of how drought stress might affect sesquiterpenoid and triterpenoid biosynthetic pathways in *A. chinensis*.

**Supplementary Information:**

The online version contains supplementary material available at 10.1186/s12870-024-04780-8.

## Background

*Atractylodes chinensis* (DC) Koidz., known as “Bei Cang Zhu” in Chinese, is mainly distributed in semiarid areas of northern China and is widely distributed in provinces such as Hebei, Inner Mongolia, Liaoning, and Shanxi [1, 2]. The rhizome of *A. chinensis* is known as traditional Chinese medicine and is rich in a series of bioactive compounds, such as sesquiterpenes, polyacetylenes, oleanolic acid, and 5-hydroxymethyl furaldehyde [3]. *Atractylodes chinensis* is used to treat digestive disorders, rheumatic diseases, and night blindness [4] due to its high levels of sesquiterpene compounds, such as atractylodin, β-eudesmol, and hinesol [5]. Terpenoids are plant secondary metabolites synthesized sequentially from a C5 isopentenyl diphosphate unit (IPP) via the mevalonic acid (MVA) pathway in the cytosol and methylerythritol phosphate (MEP) pathway in the plastids. A previous study indicated that cytosolic IPP serves as a precursor of farnesyl diphosphate (FPP) for sesquiterpenes [6]. Then, FPP is catalyzed toward two biosynthetic pathway branches: the sesquiterpenoid metabolism pathway and the triterpenoid metabolism pathway [7].

Natural populations of *A. chinensis* are currently rapidly depleting due to overharvesting and the weak reproductive capacity of perennial herbs. The artificial cultivation area for this species increases annually in northern China. A crucial question is how to ensure the survival rate during plant growth, especially at the seedling stage. *Atractylodes chinensis* is a hypogeal plant, which means that the sowing depth cannot exceed 2 cm [8]. The first true leaf emerges from the soil 15–20 days after sowing [9] and grows extremely slowly. At this time, there are only 1–2 seedling roots. Moreover, *A. chinensis* often faces severe drought stress at the seedling stage and throughout its growth period because the main production areas in China (Hebei, Inner Mongolia, Liaoning, and Shanxi provinces) have a continental monsoon climate with aridity and little rain in spring and high temperatures in summer.

Among abiotic stressors, drought stress is a major issue in northern China. The shortage of water resources seriously hampers the development, production, and quality of medicinal plants. To achieve a plant defense system in response to drought stress, a series of morphological, physiological, biochemical, and secondary metabolite responses are induced [10, 11]. The content of primary metabolites, such as soluble carbohydrates, crude protein, and proline, increases when plants are exposed to drought stress [12, 13], and the activities of antioxidant enzymes, including superoxide dismutase (SOD, EC 1.15.1.1), peroxidase (POD, EC 1.11.1.7), catalase (CAT, EC 1.11.1.6), and ascorbate peroxidase (APX, EC 1.11.1.11), are affected by drought stress [14, 15]. These physiological changes induced by drought stress are accompanied by responses of gene and metabolite expression [15–18].

Transcriptomic analysis has been conducted to gain insights into the molecular mechanisms and to screen candidate genes in response to drought stress in plants [17, 18]. Upregulated genes have been reported to enhance antioxidant capacity, regulatory factors, and repressors of premature senescence under drought stress [19]. Genes involved in the protection system against reactive oxygen species, chaperones, transcription factors, and secondary metabolism are upregulated by drought stress [20]. Drought stress decreases morphological indices and increases pulegone and menthofuran content in *Mentha piperita* [21] and increases the biosynthesis and accumulation of bioactive compounds, such as sucrose, starch, and carotenoids, in *Polygonatum kingianum* tubers [17], phenolics and lignins in potato [22], and L-ascorbic acid in *Pugionium cornutum* [23]. Researchers have investigated the molecular mechanisms of bioactive compound biosynthesis in response to drought stress in medicinal plants. For example, in *Cynanchum thesioides*, as drought stress increased, nine genes were significantly upregulated and seven genes were significantly downregulated that were involved in succinic acid biosynthesis [24]. In *A. lancea*, 10 significantly downregulated genes encoding sesquiterpene synthase were identified [15].

*Atractylodes chinensis* has commonly been medicinally used for enriching sesquiterpenes. However, the molecular mechanism of sesquiterpenoid biosynthesis in *A. chinensis* has not been well studied [7, 25]. *Atractylodes chinensis* shows strong drought resistance due to its developed fleshy rhizome and massive fibrous roots [7]; however, it has weak drought resistance at the seedling stage due to its underdeveloped root system. Previous studies have demonstrated that the content of bioactive compounds in *A. chinensis* increases under drought stress, and the key genes in the sesquiterpenoid biosynthetic pathway are highly expressed, e.g., 3-hydroxy-3-methylglutaryl coenzyme A reductase (HMGR, EC 1.1.1.34), phosphoenolpyruvic carboxylase (EC 4.1.1.31), and acetyl-CoA C-acetyltransferase (AACT, EC 2.3.1.9) [26]. To understand the complex molecular mechanisms of *A. chinensis* seedlings in response to drought stress, an integration of physiological and transcriptomic analyses was conducted on *A. chinensis* seedlings in response to drought stress over time to identify candidate genes related to sesquiterpenoid biosynthesis that respond to drought stress. Our results provide a better understanding of the mechanisms of drought resistance and sesquiterpenoid biosynthesis under drought stress in *A. chinensis*.

## Materials and methods

### Plant materials and drought treatment

This study was performed at the glass greenhouse of Hebei Normal University of Science and Technology (39°75’ N; 119°21’ E), Qinhuangdao City, Hebei Province, China. The seeds were collected from artificially cultivated 4-year-old *A. chinensis* at the farm of Hebei Normal University of Science and Technology in October 2020, so no permission was needed for the collection. The formal identification of the materials as *A. chinensis* was carried out by Professor Qiaosheng Guo who works at Nanjing Agriculture University (Nanjing, Jiangsu, China). Professor Qiaosheng Guo identified the experimental materials in the present study through a comparison with specimens deposited in the Chinese Virtual Herbarium (voucher specimen ID HBNU10016398). The study protocol complied with relevant institutional, national, and international guidelines and legislation.

Seeds of *A. chinensis* were sown at a depth of approximately 2 cm in plastic plates with 50 holes (54 cm length, 28 cm width, 11 cm depth) for seedling cultivation. The plastic plates were filled with seedling nutrient media, including coir, vermiculite, and microbial flora, on March 5, 2021. It was kept in a glass greenhouse, well-watered, and covered with a straw curtain. The straw curtain was removed when the first true leaf emerged 15–20 days after sowing. One seedling was retained per hole. At the one true leaf and one shoot stage, approximately 2-month-old seedlings, we kept healthy seedlings with similar growth for drought stress treatment for 0, 3, and 9 days. Samples from one plastic plate were used as one biological replicate, and three biological replicates were performed for each treatment. For the control group (i.e., drought stress for 0 days), the sample was watered every two days and collected on the second morning after watering. In the drought stress groups, water was withheld for natural drying for 3 and 9 days. Whole seedlings from 0 day were used as the control sample, and those from the drought stress treatments for 3 and 9 days were considered as the D3 and D9 samples. Meanwhile, we collected nutrient media for soil relative water content (RWC) evolution, calculated as described in [27]. The collected samples were divided into three groups: one part was used to analyze the plant RWC and physiological index measurements, and the other two parts were immediately placed in liquid nitrogen and stored at − 80 °C for RNA-sequencing and quantitative real − time PCR (qRT‒PCR) analysis.

### Relative water content and physiological index measurements under drought stress

A total of five whole seedlings were collected from each treatment as five biological replicates for analyzing the plant RWC of *A. chinensis*. The method was performed according to [28]. The soluble sugar content was measured using the colorimetric method with a plant soluble sugar content test kit (catalog No. A145-1-1). The crude protein content was determined using the Coomassie brilliant blue method with a total protein quantitative assay kit (catalog No. A107-1-1). The malondialdehyde (MDA) content was performed using thiobarbituric acid method with a malondialdehyde assay kit (catalog No. A003-1-1). The SOD activity was measured using the hydroxylamine method with a total superoxide dismutase assay kit (catalog No. A001-1-1). The POD activity was determined using the colorimetry method with a plant peroxidase assay kit (catalog No. A084-3-1), and CAT activity was determined using the ammonium molybdenum acid method with a hydrogen peroxide assay kit (catalog No. A011-1-1). All kits for assessing soluble sugar, crude protein, and MDA content, and SOD, POD, and CAT activity were produced by the Nanjing Jiangcheng Bioengineering Institute, Nanjing, China. Samples from five whole plants were pooled as one biological replicate for physiological index analysis, and three biological replicates were performed for each treatment.

The mean value was calculated among three biological replicates for each physiological index. Statistically significant differences in the physiological index between the control and drought treatments were determined based on Student’s t-test using SPSS version 26 software (SPSS, Chicago, USA), with *P* < 0.05 (*) and *P* < 0.01 (**) as the thresholds for significance.

### *Atractylodes chinensis* rhizome transcriptome profile and gene functional annotation under drought stress

Five rhizomes were pooled as one biological replicate for RNA extraction and three biological replicates were performed for each treatment. Total RNA from the control, D3, and D9 of *A. chinensis* were acquired separately using TRIzol reagent (Invitrogen, Carlsbad, California, USA). RNA quality control was performed as described in our previous study [7]. *Atractylodes chinensis* RNA-seq transcriptome libraries were prepared using an Illumina TruSeqTM RNA sample preparation kit (San Diego, CA). According to the manufacturer’s instructions (Invitrogen, Carlsbad, California, USA), genomic DNA was removed using DNaseI (TaKaRa, Osaka, Japan). Then, the integrity and purity of the total RNA quality were determined by a 2100 Bioanalyzer (Agilent Technologies, Inc., Santa Clara CA, USA) and quantified using an ND-2000 (NanoDrop Thermo Scientific, Wilmington, DE, USA). Only high-quality RNA samples (OD260/280 = 1.8–2.2, OD260/230 ≥ 2.0, RIN ≥ 8.0, 28 S:18 S ≥ 1.0, > 1 µg) were used to construct the sequencing library. RNA purification, reverse transcription, library construction and Illumina sequencing of *A. chinensis* were performed at Shanghai Majorbio Biopharm Biotechnology Co., Ltd. (Shanghai, China), according to the manufacturer’s instructions (Illumina, San Diego, CA). Transcriptome libraries of *A. chinensis* were prepared using an Illumina TruSeqTM RNA sample preparation kit (San Diego, CA). Poly(A) mRNA was purified from total RNA using oligo-dT-attached magnetic beads and then fragmented by the fragmentation buffer. Taking these short fragments as templates, double-stranded cDNA was synthesized using a SuperScript double-stranded cDNA synthesis kit (Invitrogen, Carlsbad, California, USA) with random hexamer primers. Then, the synthesized cDNA was subjected to end-repair, phosphorylation, and ‘A’ base addition according to Illumina’s library construction protocol. Libraries were selected for cDNA target fragments of 200–300 bp on 2% Low Range Ultra Agarose followed by PCR amplification using Phusion DNA polymerase (New England Biolabs, Boston, MA) for 15 PCR cycles. After quantification by TBS380, three RNA–seq libraries were sequenced in a single lane on an Illumina NovaSeq 6000 sequencer (Illumina, San Diego, CA) for 2 × 150 bp paired-end reads. The raw paired–end reads were trimmed and quality controlled by SeqPrep (https://github.com/jstjohn/SeqPrep) and Sickle (https://github.com/najoshi/sickle) with default parameters. Then clean transcriptome data from *A. chinensis* were used for de novo assembly with Trinity (http://trinityrnaseq.sourceforge.net/).

The transcriptome data generated in this study have been uploaded to the NCBI (SAMN33016714) Sequence Read Archive: PRJNA930596 (https://dataview.ncbi.nlm.nih.gov/object/PRJNA930596?reviewer=strmegng5mpqeqdt167hhn6i5g). All assembled transcripts were searched against the NCBI protein nonredundant (NR Version 2020.06, https://www.ncbi.nlm.nih.gov/public/), Clusters of Orthologous Groups of Proteins (COG, http://eggnogdb.embl.de/#/app/home), Kyoto Encyclopedia of Genes, Genomes (KEGG Version 2020.07, http://www.genome.jp/kegg/), Pfam (Version 33.1, http://pfam.xfam.org/), and Swiss-prot (Version 2020.06, ftp://ftp.uniprot.org/pub/databases/UniProt/current_release/knowledgebase/complete/uniprot_sprot.fasta.gz) databases using BLASTX to identify the proteins with the highest sequence similarity with the given transcripts to retrieve their functional annotations. A typical cut-off E-value less than 1.0 × 10^− 5^ was set. The BLAST2GO (Version 2020.0628, http://www.blast2go.com/b2ghome) program was used to obtain GO annotations of assembled transcripts to describe biological processes, molecular functions and cellular components.

### Identification of differentially expressed genes under drought stress

To identify differentially expressed genes (DEGs) between the control and drought stress treatments, the expression level of each transcript was calculated according to the transcript per million reads method. RNA-seq by expectation-maximization (http://deweylab.biostat.wisc.edu/rsem/) was used to quantify gene abundance. Differential expression analysis was performed using DESeq2 with *P*adjust < 0.05 and |log2FC|≥1 as the thresholds for significant DEGs. In addition, functional enrichment analysis, including GO and KEGG, was performed to identify which DEGs were significantly enriched in GO terms and KEGG metabolic pathways at a Bonferroni-corrected *P*-value ≤ 0.05 compared with the whole transcriptome background. GO functional enrichment and KEGG pathway enrichment analyses were carried out using Goatools (https://github.com/tanghaibao/Goatools) and KOBAS (http://kobas.cbi.pku.edu.cn/home.do), respectively.

### RNA extraction and quantitative real-time PCR

The qRT‒PCR primers for the 15 selected DEGs involved in sesquiterpenoid and triterpenoid biosynthetic pathways were designed using Primer3 software (Table S1). Total RNA was extracted from the control, D3, and D9 individually using a TRIzol RNA isolation reagent (Sigma-Aldrich, St Louis, USA) following the manufacturer’s instructions.

For the qRT‒PCR assay, the isolated total RNA of the control, D3, and D9 was treated with DNase I to remove the genomic DNA, and cDNA was synthesized using a cDNA Synthesis SuperMix Kit (TransGen Biotech, Beijing, China). qRT‒PCR was performed on an ABI 7500 Fast Real-Time System (Applied Biosystems, New York, USA) using TransStart Top Green qPCR Supermix (TransGen Biotech, Beijing, China) to verify the transcript levels of the 15 selected DEGs. The qRT‒PCR reactions and amplifications were performed as described in [29]. As an internal control, the *UBQ2* gene was used to calculate the relative expression level [7]. The relative expression of the 15 selected DEGs was calculated using the 2 ^−∆Ct^ method.

## Results

### Phenotypic and physiological changes in *A. chinensis* during drought stress

**Fig. 1:**
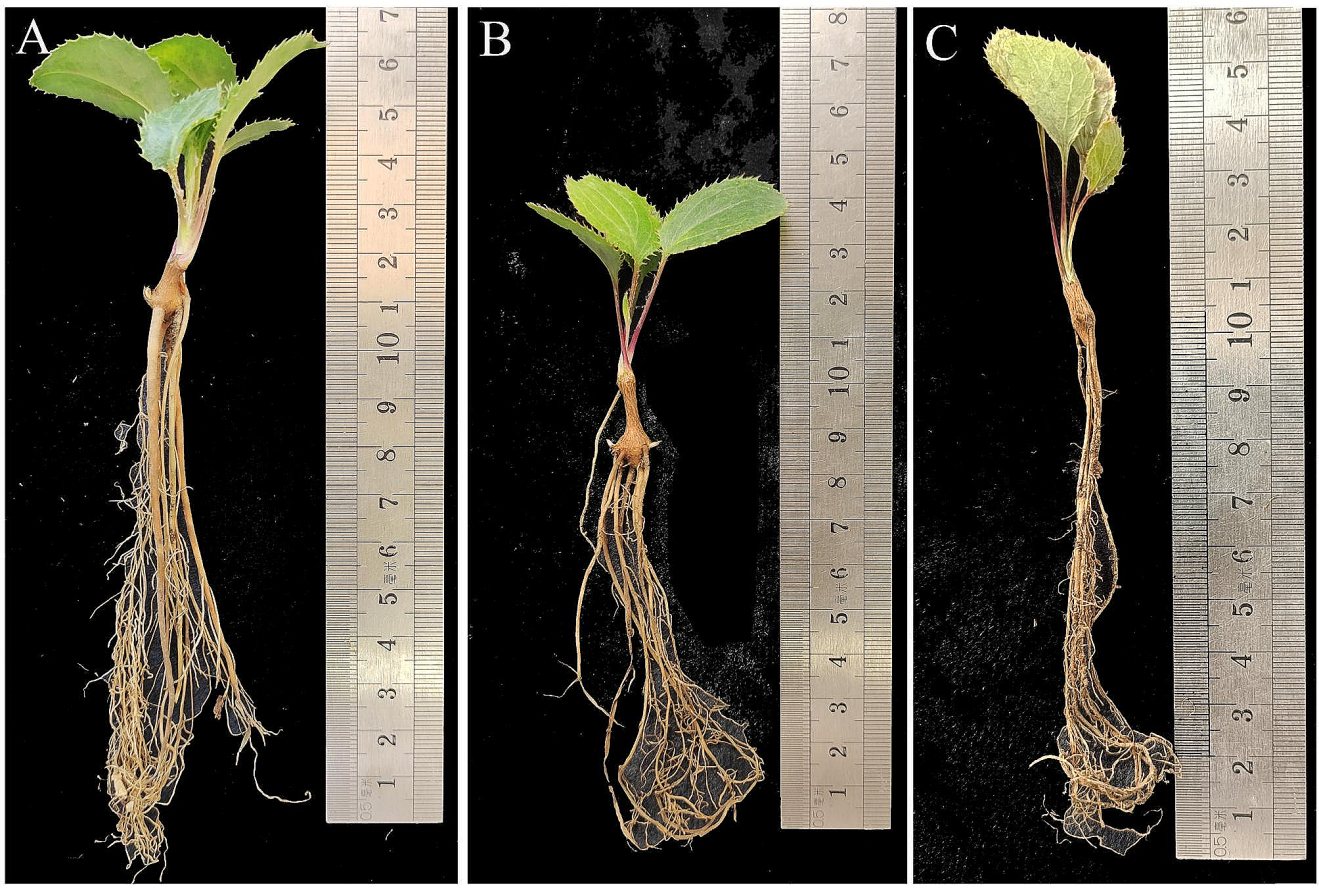
Plant phenotypic characterization of *Atractylodes chinensis* under drought stress. (**A**) control; (**B**) D3; (**C**) D9

Upon drought stress prolongation, more plants exhibited symptoms of dehydration, with yellowing or even dried leaves. At D3 (Fig. 1B), the phenotype of *A. chinensis* seedlings showed no significant difference from the control (Fig. 1A), but a greater percentage of yellowing or dried leaves was found at D9 (Fig. 1C).

**Fig. 2:**
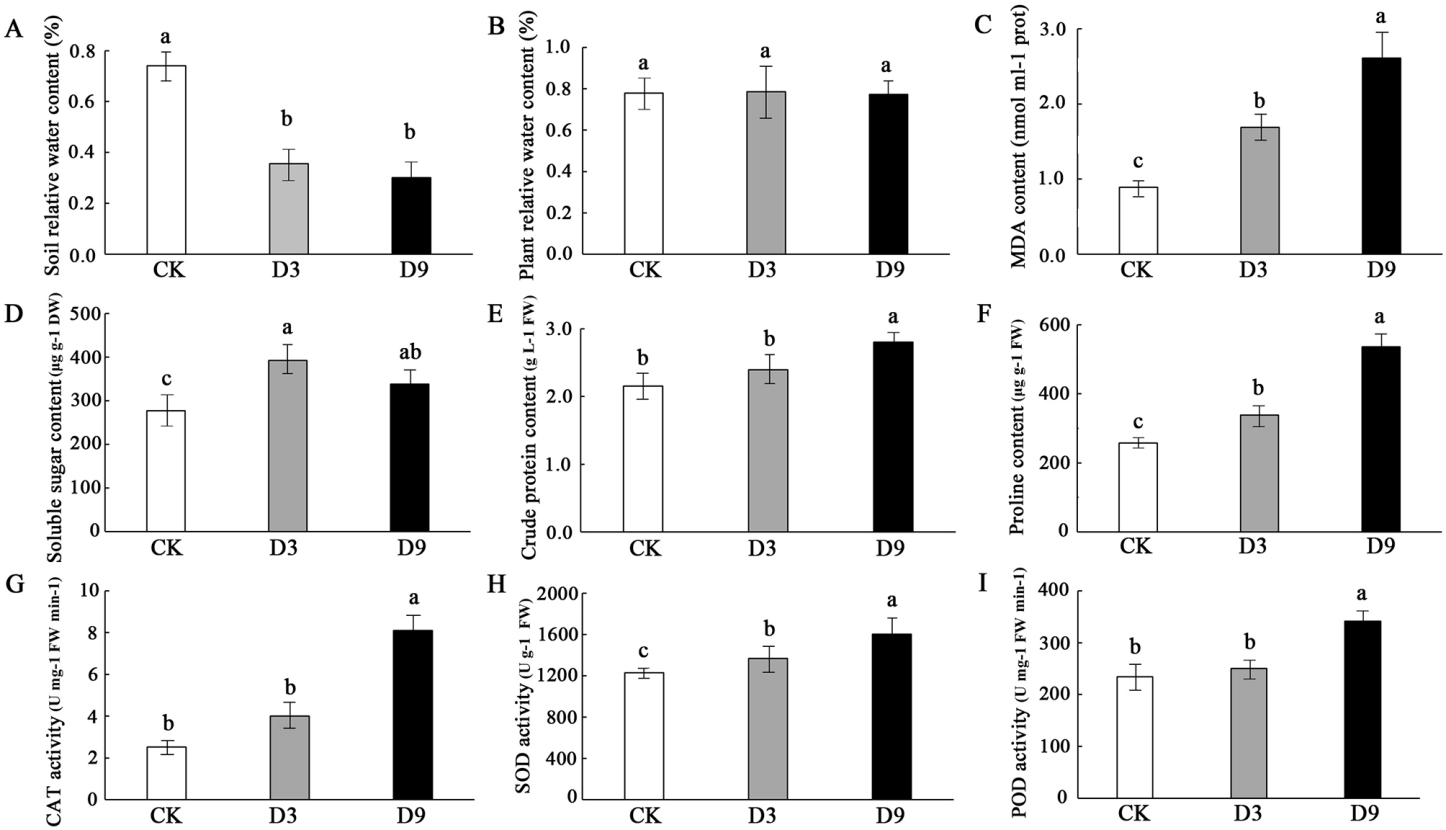
Effects of drought stress on the relative water content of soil and physiological parameters of roots in *Atractylodes chinensis*. Control; drought stress for 3 days (D3); drought stress for 9 days (D9). Values represent mean ± SE (*n* = 3). Letters indicate significant differences among control, D3, and D9 at *P* < 0.05 based on one-way ANOVA. (**A**) Soil relative water content; (**B**) Plant relative water content; (**C**) MDA content; (**D**) Soluble sugar content; (**E**) Crude protein content; (**F**) Proline content; (**G**) CAT activity; (**H**) SOD activity; (**I**) POD activity

To analyze the physiological changes in *A. chinensis* subjected to drought stress, we evaluated eight physiological indices from the control, D3, and D9. With prolonged drought stress, the soil RWC significantly decreased from 74.40 to 35.54% in D3 (*P* < 0.05), and then gradually changed to 30.09% in D9 (Fig. 2A). The whole plant RWC of the drought stress treatments displayed no significant difference from that of the control (Fig. 2B). The MDA content indicates free radical formation and membrane damage in plants under stress. The MDA content increased significantly with prolonged drought stress (*P* < 0.05) and was more than two times higher in D9 than in the control (Fig. 2C). The soluble sugar content was significantly increased in drought-stressed plants of D3 and D9 compared with control plants (*P* < 0.05, Fig. 2D). The crude protein content changed similarly to CAT and POD activity (Fig. 2E and G, and 2I). Compared with the control, the crude protein content and the antioxidant enzyme activity of CAT and POD were significantly increased in D9 (*P* < 0.05) but showed no significant difference with D3. The proline content and antioxidant enzyme SOD activity were increased significantly with prolonged drought stress (*P* < 0.05, Fig. 2F and H). These results suggest that *A. chinensis* maintains plant RWC and changes physiological parameters to adapt to drought stress, which could maintain intracellular oxidative homeostasis.

### Transcriptomic profiles of *A. chinensis* in response to drought stress

RNA-seq analysis was conducted in the control, D3, and D9 to further reveal the molecular changes in *A. chinensis* when exposed to drought stress. A summary of the *A. chinensis* RNA-seq statistics is presented in Table 1. Samples belonging to the control, D3, and D9 generated 6.28, 6.30, and 6.33 Gb of read data, respectively, with an average Q30 of 94.59%. We obtained 215,665 unigenes with an average length of 759.09 bp and an N50 of 1140 bp. These results indicate that the sequencing data quality was high and could be used for further analysis.

**Table 1:** Statistics of RNA-seq for the control and drought-treated samples of *Atractylodes chinensis*

Samples	Raw reads (M)	Clean bases (Gb)	Clean reads (bp)	Error rate (%)	Q30 (%)	GC (%)
control	43,327,492	6.28	21,440,251	2.46	94.59	45.14
D3	44,048,600	6.30	21,713,348	2.46	94.59	45.24
D9	44,174,204	6.33	21,817,122	2.46	94.58	45.35
Number of unigenes			215,665			
Average length of unigenes (bp)			759.09			
N50 of unigenes (bp)			1,140			

Note: D3 drought stress for 3 days, D9 drought stress for 9 days

**Fig. 3:**
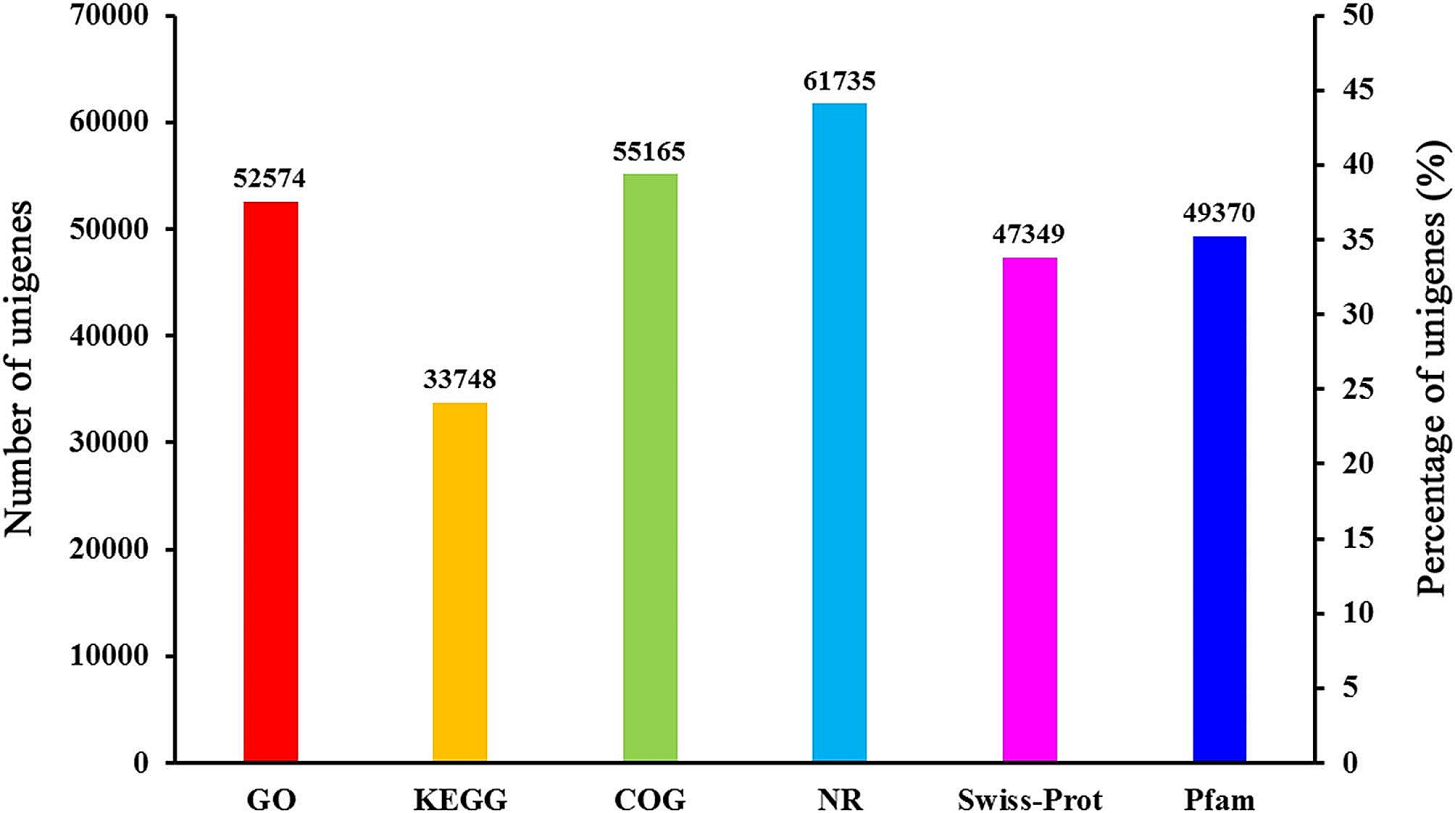
Statistics of annotation for unigenes of *Atractylodes chinensis* in six public databases

A total of 130,195 unigenes were annotated to one or more public databases. Of them, 52,574 unigenes (40.38% of the total assembled unigenes) had matches in the GO database, 33,748 (25.92%), 55,165 (42.37%), 61,735 (47.42%), 47,349 (36.37%), and 49,370 (37.92%) unigenes showed significant similarity to sequences in the KEGG, COG, NR, Swiss-Prot, and Pfam databases, respectively (Fig. 3). In total, 70,221 unigenes (32.56%) showed high similarity to sequences in all six public databases.

### Differentially expressed gene analysis of *A. chinensis* in response to drought stress

**Fig. 4:**
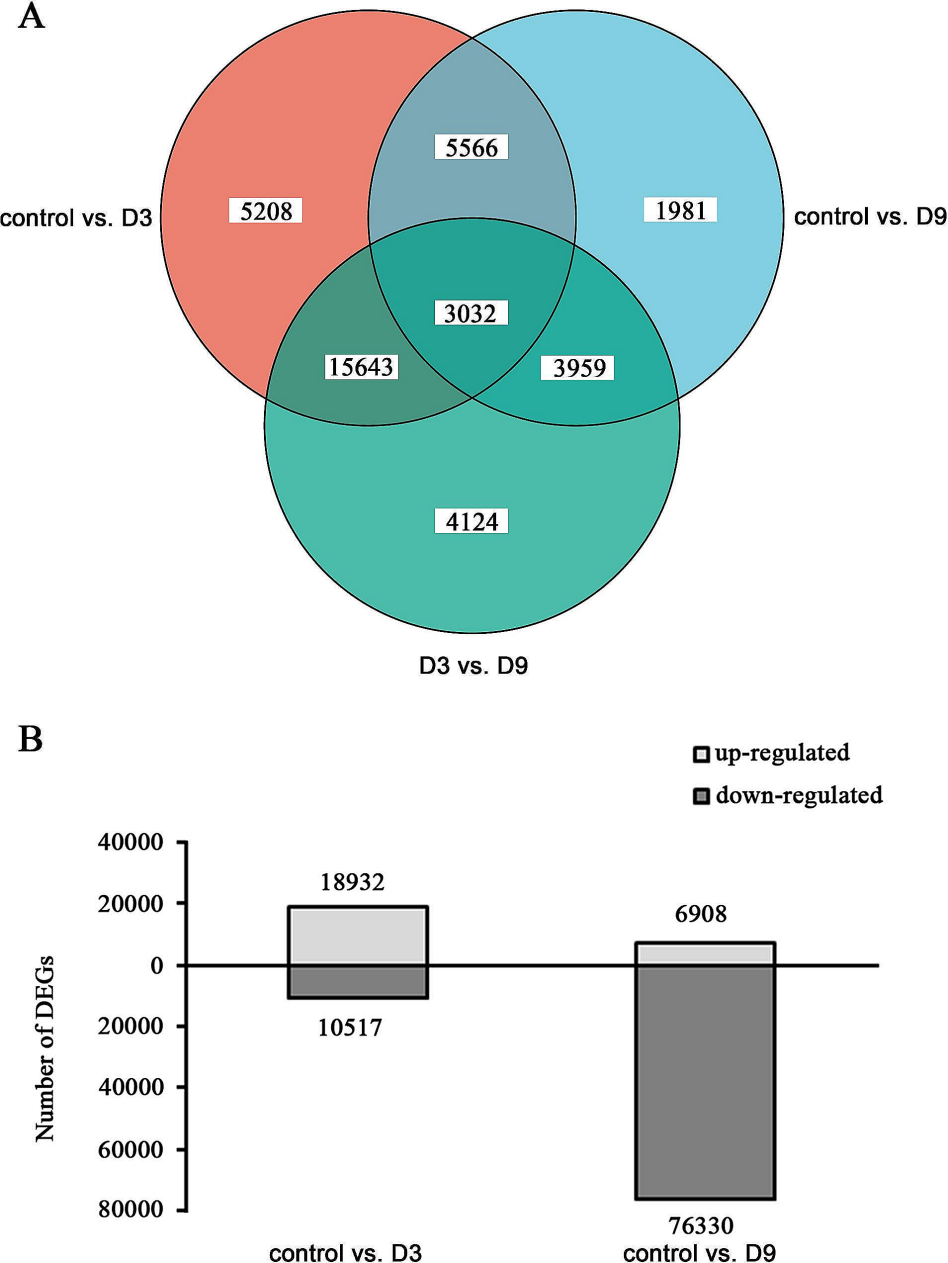
Venn diagram and differential gene expression under drought stress in the comparison between the control and D3 (control vs. D3) and between the control and D9 (control vs. D9). (**A**) Venn diagram of unigenes from control, D3, and D9 of *Atractylodes chinensis*; (**B**) Differential gene expression under drought stress in comparisons of control vs. D3 and control vs. D9

To gain a deeper understanding of the molecular mechanisms underlying the physiological changes in *A. chinensis* under drought stress, DGEs were identified (Fig. 4A). A total of 14,538 DEGs were screened (Table S2). A total of 3,032 unigenes were shared by all three groups, and 5208, 1981, and 4124 unigenes were specific to the control, D3, and D9, respectively. As shown in Fig. 4B, more DEGs were discovered in the comparison between the control and D9 (control vs. D9) than between the control and D3 (control vs. D3). There were more upregulated unigenes than downregulated unigenes in control vs. D3 in contrast to control vs. D9. A total of 29,449 DEGs were detected in control vs. D3 with 18,932 upregulated unigenes and 10,517 downregulated unigenes, and 83,238 DEGs were found in control vs. D9 with 6908 upregulated and 76,330 downregulated unigenes.

**Fig. 5:**
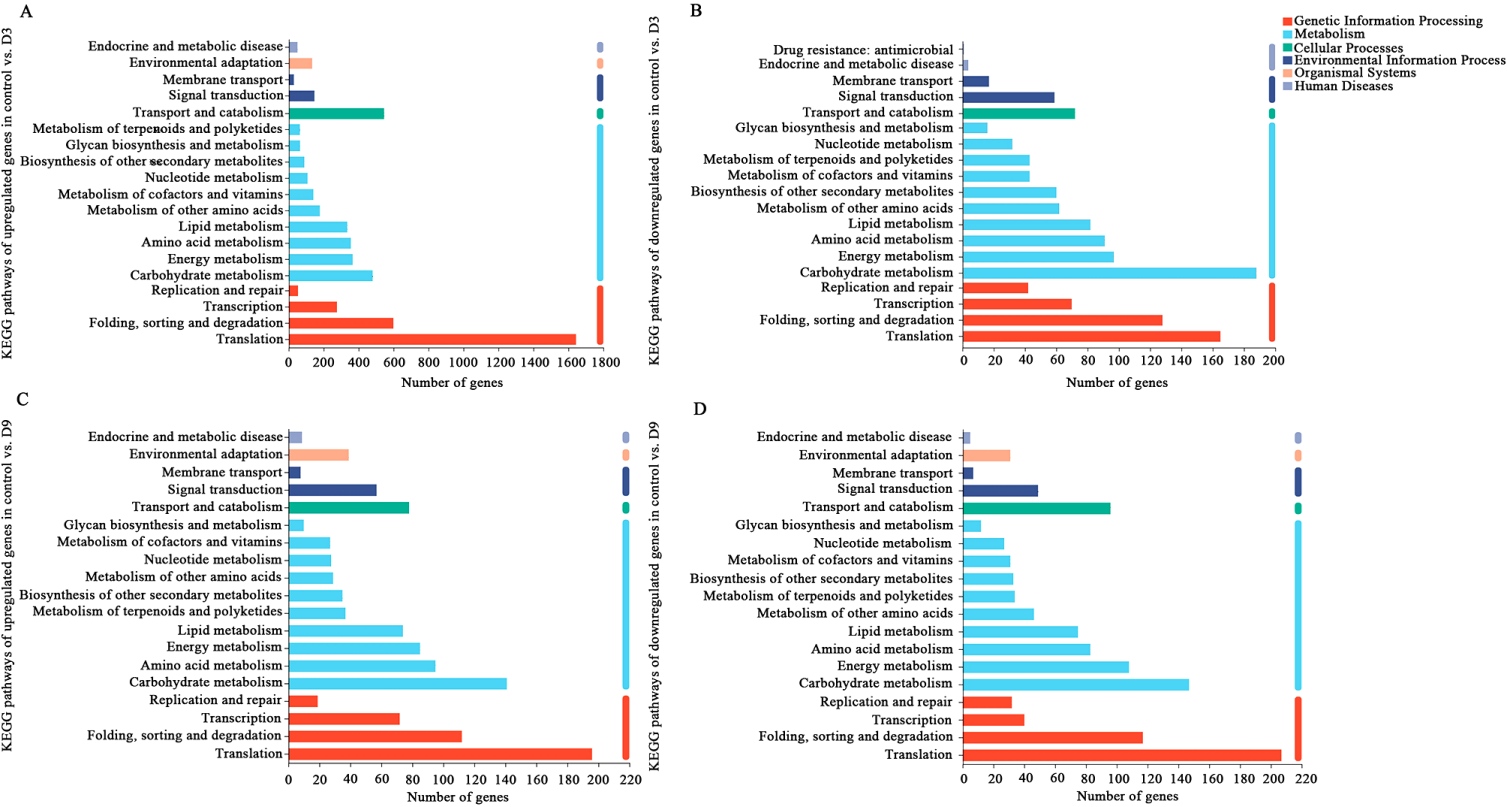
KEGG classification of up- and downregulated DEGs in the comparison between the control and D3 (control vs. D3) and between the control and D9 (control vs. D9). (**A**) Upregulated DEGs in control vs. D3; (**B**) Down-regulated DEGs in control vs. D3; (**C**) Up-regulated DEGs in control vs. D9; (**D**) Down-regulated DEGs in control vs. D9

KEGG pathway classification was performed to classify the functions of the DEGs in control vs. D3 and control vs. D9. The DEGs in control vs. D3 were assigned to 131 KEGG pathway classifications (Table S3), and 126 KEGG pathways were assigned to control vs. D9 (Table S4). For the 18,932 upregulated unigenes in control vs. D3, most of the upregulated unigenes were classified as translation (1644 unigenes), followed by folding, sorting and degradation (600 unigenes), and transport and catabolism (545 unigenes, Fig. 5A). The 10,517 downregulated unigenes in control vs. D3 were mainly involved in several categories, including carbohydrate metabolism with 188 unigenes, translation with 165 unigenes, and folding, sorting and degradation with 128 unigenes (Fig. 5B). There were 64 upregulated and 43 downregulated unigenes annotated into terpenoid and polyketide metabolism. In addition, 90 upregulated and 60 downregulated unigenes were assigned to the biosynthesis of other secondary metabolites. In the terpenoid and polyketide metabolism, terpenoid backbone biosynthesis had the highest number of unigenes (21 upregulated and five downregulated unigenes), followed by limonene and pinene degradation (16 upregulated and eight downregulated unigenes), sesquiterpenoid and triterpenoid biosynthesis (seven upregulated and five downregulated unigenes), and carotenoid biosynthesis (five upregulated and seven downregulated unigenes).

For the DEGs in control vs. D9, the majority of annotated unigenes fell into three KEGG categories: translation (196 upregulated and 207 downregulated unigenes), carbohydrate metabolism (141 upregulated and 147 downregulated unigenes), and folding, sorting and degradation (112 upregulated and 127 downregulated unigenes, Fig. 5C and D). There were 37 upregulated and 34 downregulated unigenes related to terpenoid and polyketide metabolism. A total of 35 upregulated and 33 downregulated unigenes were annotated for the biosynthesis of other secondary metabolites. In the terpenoid and polyketide metabolism, terpenoid backbone biosynthesis had the highest number of unigenes (11 upregulated and five downregulated unigenes), followed by sesquiterpenoid and triterpenoid biosynthesis (six upregulated and six downregulated unigenes), diterpenoid biosynthesis (two upregulated and eight downregulated unigenes), and carotenoid biosynthesis (six upregulated and four downregulated unigenes).

### GO functional enrichment and KEGG pathway enrichment analysis of DEGs

**Fig. 6:**
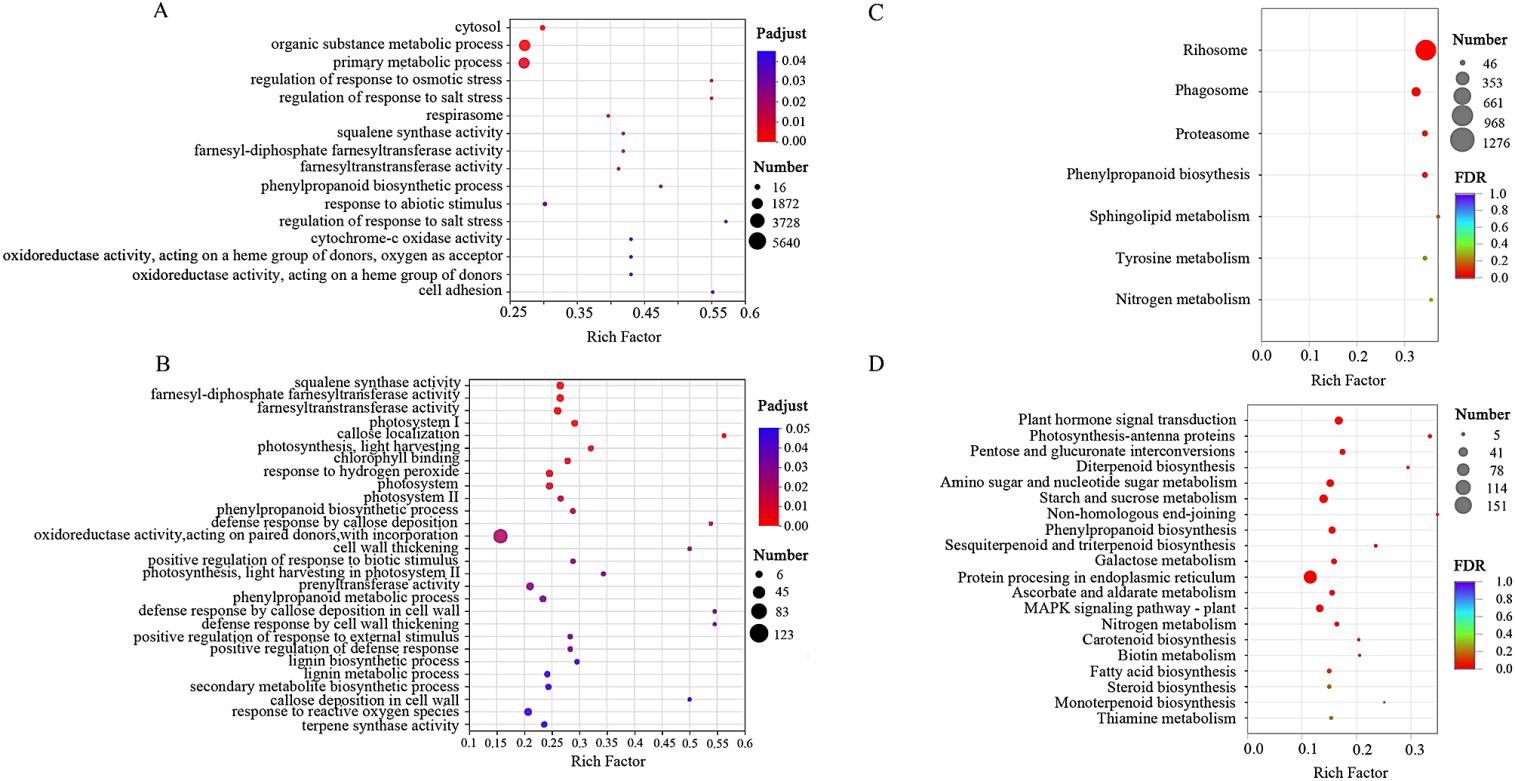
Scatter chart displaying GO classifications and KEGG pathway enrichment of DEGs between the control and different drought-treated *Atractylodes chinensis*. (**A**) Top 20 GO enrichment classifications in control vs. D3; (**B**) Top 20 GO enrichment classifications in control vs. D9; (**C**) KEGG enrichment pathways in control vs. D3; (**D**) Top 20 KEGG enrichment pathways in control vs. D9. The y-axis presents the GO classifications or KEGG pathways, and the x-axis presents the rich factors. Dot size corresponds to the number of distinct genes, whereas dot colour reflects the *P*adjust

The DEGs in both control vs. D3 and control vs. D9 were subjected to enrichment analysis based on GO terms and KEGG pathways (Fig. 6) to gain further insight into the biological functions of DEGs in response to drought stress. We specifically focused on the GO enrichment categories for screening the drought-related categories, and 156 GO categories were significantly enriched in the comparison control vs. D3 (Table S5). The first three enrichment categories included organic substance metabolic process (5640 unigenes), primary metabolic process (5304 unigenes), and cytosol (819 unigenes; Fig. 6A). In addition, stress-related processes (regulation of response to salt stress, response to abiotic stimulus, and positive regulation of response to salt stress), oxidoreductase activity (oxidoreductase activity, acting on a haem group of donors, oxygen as acceptor and oxidoreductase activity, and acting on a haem group of donors), cytochrome-c oxidase activity, and regulation of response to osmotic stress were significantly enriched in control vs. D3. There were four enrichment categories related to secondary metabolites, three of which were involved in sesquiterpenoid and triterpenoid biosynthesis, including squalene synthase activity (49 unigenes), farnesyl-diphosphate farnesyltransferase activity (49 unigenes), and farnesyltransferase activity (49 unigenes). We screened 94 enriched GO categories in the comparison control vs. D9 (Table S6), 24 of which were drought-related (Fig. 6B). These 24 GO categories were classified into four groups. The first group was related to physical defense, such as cell wall thickening (7 unigenes), lignin biosynthetic process (13 unigenes), and callose localization (9 unigenes). The second group was related to physiological defense, such as positive regulation of defense response (15 unigenes), response to hydrogen peroxide (28 unigenes), and response to reactive oxygen species (31 unigenes). The third group was composed of six photosynthesis associated responses, including photosystem I (23 unigenes), photosystem (28 unigenes), photosystem II (21 unigenes), photosynthesis, light harvesting, photosynthesis (17 unigenes), light harvesting in photosystem I (11 unigenes), and chlorophyll binding (22 unigenes). The fourth group comprised eight secondary metabolite biosynthesis terms, five of which were directly related to sesquiterpenoid and triterpenoid biosynthesis, including squalene synthase activity (31 unigenes), farnesyl-diphosphate farnesyltransferase activity (31 unigenes), farnesyltransferase activity (31 unigenes), prenyltransferase activity (33 unigenes), and terpene synthase activity (21 unigenes).

The KEGG enrichment analysis of the DEGs showed time-specific results. For control vs. D3, the KEGG enrichment analysis of the DEGs indicated the enrichment of only seven metabolic processes, with the majority involved in ribosomes (1276 unigenes), followed by phagosomes (250 unigenes), proteasomes (105 unigenes), and phenylpropanoid biosynthesis (99 unigenes; Fig. 6C). Of the top 20 enriched KEGG metabolism pathways for control vs. D9, the most significantly enriched pathway was protein processing in the endoplasmic reticulum (151 unigenes), followed by starch and sucrose metabolism (63 unigenes) and plant hormone signal transduction (56 unigenes). In control vs. D9, secondary metabolites were significantly enriched with a great majority of terpenoid metabolism pathways, including sesquiterpenoid and triterpenoid biosynthesis (12 unigenes), diterpenoid biosynthesis (10 unigenes), and monoterpenoid biosynthesis (5 unigenes) (Fig. 6D). This result indicates that the prolongation of drought stress can induce the biosynthesis of secondary metabolites.

### Candidate genes involved in sesquiterpenoid biosynthesis

**Fig. 7:**
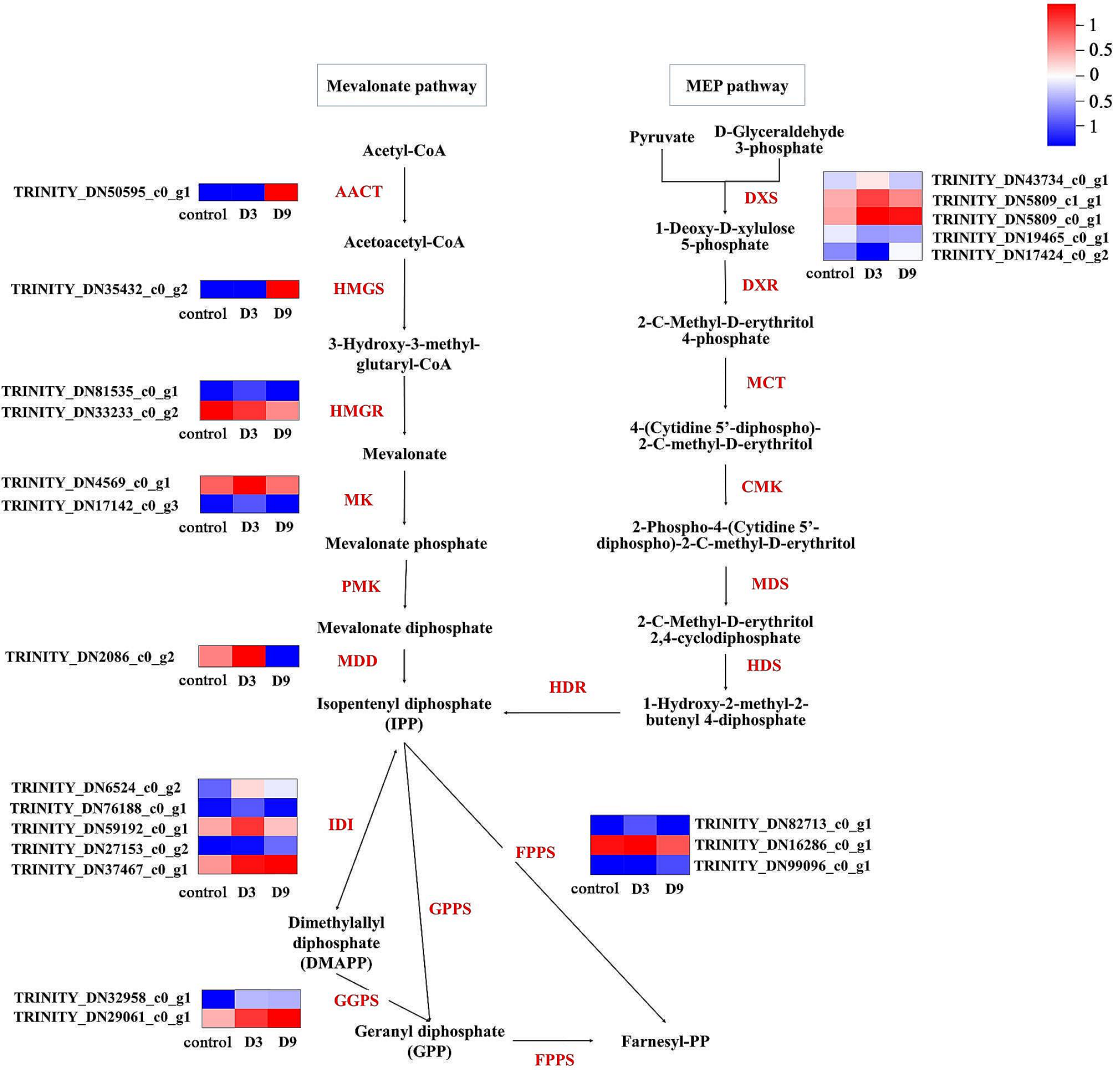
The putative terpenoid backbone biosynthetic pathway and the DEGs in *Atractylodes chinensis* response to drought stress. Note: Red letters indicate putative enzymes for the terpenoid backbone biosynthetic pathway. The progression of the colour scale from blue to red represents an increase in the TPM values

We screened 48 unigenes encoding nine known enzymes in the MVA pathway, 18 unigenes encoding four known enzymes in the MEP pathway, 38 unigenes encoding eight known enzymes in the sesquiterpenoid biosynthetic pathway, and 88 unigenes encoding eight known enzymes in the triterpenoid biosynthetic pathway in the *A. chinensis* transcriptome database in response to drought stress (Table 2). A few of these unigenes in the drought stress groups were differentially expressed compared with the control group. For the terpenoid backbone pathway, more unigene-encoding enzymes related to the MVA pathway were obtained than those encoding the MEP pathway. Similar conditions occurred in the downstream pathway. More DEGs encoding enzymes involved in the triterpenoid biosynthetic pathway were screened than in the sesquiterpenoid biosynthetic pathway (Table 2). A heatmap of DEGs involved in the terpenoid backbone biosynthetic pathway is shown in Fig. 7. Most unigenes (18 out of 22) were upregulated under drought stress. Significant differential expression of these DEGs could be recognized among the drought stress groups and the control group. Of the 22 DEGs, 16 were upregulated in D3, of which 13 were then downregulated in D9. Of the 22 DEGs, eight showed the highest expression levels at D9. The starting enzyme, AACT, was upregulated in D9, but there was no difference in D3. One of the rate-limiting enzymes, HMGR, was downregulated with the prolongation of drought stress; the other was upregulated in D3 and then downregulated in D9.

**Table 2:** Discovery of unigenes-encoding enzymes involved in terpenoid backbone biosynthesis, sesquiterpenoid and triterpenoid biosynthesis in *Atractylodes chinensis* transcriptome

Pathway	Enzymes name	EC number	Abbreviation	Total number of unigenes	Number of DEGs	
					control vs. D3	control vs. D9
MVA	Acetyl-CoA C-acetyltransferase	2.3.1.9	AACT	11	0	1
	3-hydroxy-3-methylglutaryl coenzyme A synthase	2.3.3.10	HMGS	5	0	1
	3-hydroxy-3-methylglutaryl coenzyme A reductase	1.1.1.34	HMGR	2	1	1
	mevalonate kinase	2.7.1.36	MK	4	2	0
	phosphomevalonate kinase	2.7.4.2	PMK	2	0	0
	Mevalonate 5-diphosphate decarboxylase	4.1.1.33	MDD	3	0	1
	Isopentenyl-diphosphate delta-isomerase	5.3.3.2	IDI	11	3	2
	Geranylgeranyl pyrophosphate synthase	2.5.1.29	GGPS	5	2	0
	Farnesyl diphosphate synthase	2.5.1.1 2.5.1.10	FPPS	3	1	2
MEP	1-deoxy-D-xylulose-5-phosphate synthase	2.2.1.7	DXS	11	4	1
	1-deoxy-D-xylulose-5-phosphate reductoisomerase	1.1.1.267	DXR	4	0	0
	2-c-methyl-d-erythritol 2,4-cyclodiphosphate synthase	4.6.1.12	MDS	1	0	0
	4-diphosphocytidyl-2-C-methyl-D-erythritol kinase	2.7.1.148	CMK	2	0	0
Sesquiterpenoid	Germacrene D synthase	4.2.3.75	GDS	1	0	0
	Germacrene A synthase	4.2.3.23	GAS	5	0	0
	beta-caryophyllene synthase	4.2.3.57	QHS	17	0	0
	Farnesol dehydrogenase	1.1.1.354	FLDH	1	0	0
	Nerolidol synthase	4.2.3.48	NS	2	0	1
	alpha-farnesene synthase	4.2.3.46	AFS	3	0	1
	Terpene synthase	4.2.3.49 4.2.3.47 3.1.7.6	TPS	8	3	0
Triterpenoid	Squalene synthase	1.3.1.96	SQS	6	2	0
	Squalene epoxidase	1.14.14.17	SE	4	2	0
	Dammarenediol-II synthase	4.2.1.125	DS	13	4	4
	Beta-amyrin synthase	5.4.99.39	β-AS	31	2	3
	Lupeol synthase	5.4.99.41	LUS	1	0	0
	Lanosterol synthase	5.4.99.7	LAS	31	0	0
	Geraniol synthase	3.1.7.11	GS	1	1	0
	Cytochrome P450	1.14.14.1	CYP450	1	0	1

Several key genes involved in the sesquiterpenoid and triterpenoid biosynthetic pathways were discovered by DEG analysis in the comparisons control vs. D3 and control vs. D9 (Table 3). A total of 12 unigenes were discovered in control vs. D3 coding five key enzymes, and 12 unigenes were discovered in control vs. D9 coding seven key enzymes. The expression patterns of the DEGs encoding sesquiterpenoid and triterpenoid metabolism-related enzymes were explored. Compared with the control, we found seven upregulated and five downregulated unigenes involved in the sesquiterpenoid and triterpenoid biosynthetic pathway for control vs. D3 and six upregulated and six downregulated unigenes for control vs. D9. Among these unigenes, eight unigenes appeared in both comparisons of control vs. D3 and control vs. D9 and showed the same regulation patterns. In further analysis of these eight unigenes, we paid close attention to TRINITY_DN833_c0_g1, encoding dammarenediol II synthase (DS), which was the most upregulated expression during drought stress, followed by TRINITY_DN81522_c0_g1 encoding lanosterol synthase (LAS). The lowest downregulated expression was observed in TRINITY_DN1332_c1_g1 encoding squalene epoxidase (SE), followed by TRINITY_DN29281_c0_g1 encoding DS. These eight unigenes may be candidate genes for *A. chinensis* in response to drought stress. Focusing on the DEGs related to sesquiterpenoid and triterpenoid biosynthetic pathways, we also discovered eight specifically expressed transcripts in D3 and D9.

**Table 3:** Regulation, relative expression and annotation of DEGs related to sesquiterpenoid and triterpenoid biosynthetic pathways in comparisons of control vs. D3 and control vs. D9

Comparison	Transcript ID	Regulation	Log2FC	Abbreviation	Annotation
control vs. D3	TRINITY_DN626_c0_g1	up	1.12	SE2	Squalene epoxidase
	TRINITY_DN5420_c0_g1	up	1.38	AS1	beta-amyrin synthase
	TRINITY_DN4574_c0_g1	up	1.49	AS3	beta-Amyrin synthase
	TRINITY_DN3403_c1_g2	up	1.44	GS	Geraniol synthase
	TRINITY_DN3072_c1_g1	up	2.47	DS1	Dammarenediol II synthase
	TRINITY_DN833_c0_g1	up	6.93	DS4	Dammarenediol II synthase
	TRINITY_DN81522_c0_g1	up	4.35	DS3	Dammarenediol II synthase
	TRINITY_DN29281_c0_g1	down	-6.40	DS2	Dammarenediol II synthase
	TRINITY_DN2734_c0_g1	down	-2.31	SQS1	Squalene synthase
	TRINITY_DN1332_c1_g1	down	-6.53	SE1	Squalene epoxidase
	TRINITY_DN10738_c0_g2	down	-1.22	DS5	Dammarenediol II synthase
	TRINITY_DN11881_c0_g2	down	-4.87	SQS2	Squalene synthase
control vs. D9	TRINITY_DN4574_c0_g3	up	1.65	AS2	beta-Amyrin synthase
	TRINITY_DN4574_c0_g1	up	1.02	AS3	beta-Amyrin synthase
	TRINITY_DN3403_c1_g2	up	1.38	GS	Geraniol synthase
	TRINITY_DN3072_c1_g1	up	2.14	DS1	Dammarenediol II synthase
	TRINITY_DN833_c0_g1	up	5.66	DS4	Dammarenediol II synthase
	TRINITY_DN81522_c0_g1	up	4.10	DS3	Dammarenediol II synthase
	TRINITY_DN29281_c0_g1	down	-3.58	DS2	Dammarenediol II synthase
	TRINITY_DN2734_c0_g1	down	-1.23	SQS1	Squalene synthase
	TRINITY_DN1332_c1_g1	down	-10.16	SE1	Squalene epoxidase
	TRINITY_DN1163_c1_g1	down	-1.03	P450	Cytochrome P450
	TRINITY_DN30423_c0_g1	down	-1.39	NS	(3 S,6E)-nerolidol synthase
	TRINITY_DN7063_c0_g3	down	-1.14	AS4	beta-Amyrin synthase

**Fig. 8:**
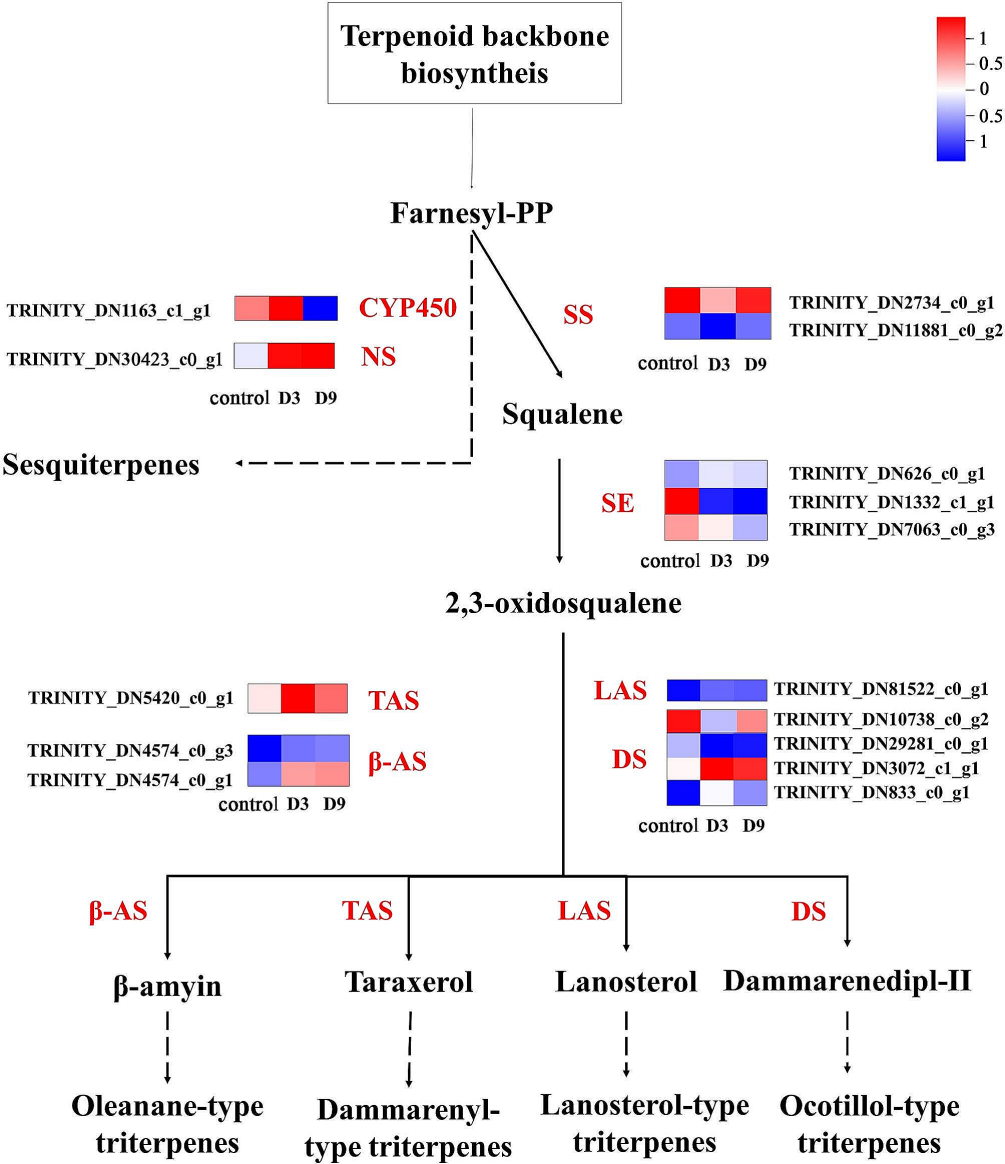
Enzymes involved in putative sesquiterpenoid and triterpenoid biosynthetic pathways in *Atractylodes chinensis* responses to drought stress. Note: Red letters indicate putative enzymes for the action of sesquiterpenoid and triterpenoid biosynthetic pathways. The progression of the colour scale from blue to red represents an increase in the TPM values. The solid line represents a direct catalytic reaction, and the dotted line represents an indirect catalytic reaction

A heatmap of 15 DEGs related to the sesquiterpenoid and triterpenoid biosynthetic pathways is presented in Fig. 8. Two unigenes encoding the first key enzyme, squalene synthase (SQS), in the triterpenoid biosynthetic pathway were downregulated in the early stage of drought stress (D3) and then upregulated under the prolongation of drought stress (D9). The second enzyme was SE in the triterpenoid biosynthetic pathway, with three DEGs responsible, one of which was opposite the expression of SQS, and the other two DEGs were downregulated under drought stress. There were four DEGs encoding DS, two of which were upregulated in D3 and then downregulated in D9. The other two DEGs showed the opposite pattern. The downstream enzymes related to triterpenoid biosynthesis, including two beta-amyrin synthase genes (*AS*), one lanosterol synthase genes (*LAS*), and one taraxerol synthase genes (*TAS*), were upregulated in D3. The *LAS* and *TAS* were then downregulated in D9. Only two sesquiterpenoid compound biosynthesis-responsible genes were screened under drought stress, including the cytochrome P450 gene (*CYP450*) and the (3 S,6E)-nerolidol synthase gene (*NS*). The NS encoding gene was downregulated under drought stress. The CYP450 encoding gene was first upregulated in D3 and then downregulated in D9.

### Validation of the expression patterns of 16 screened DEGs by qRT‒PCR

**Fig. 9:**
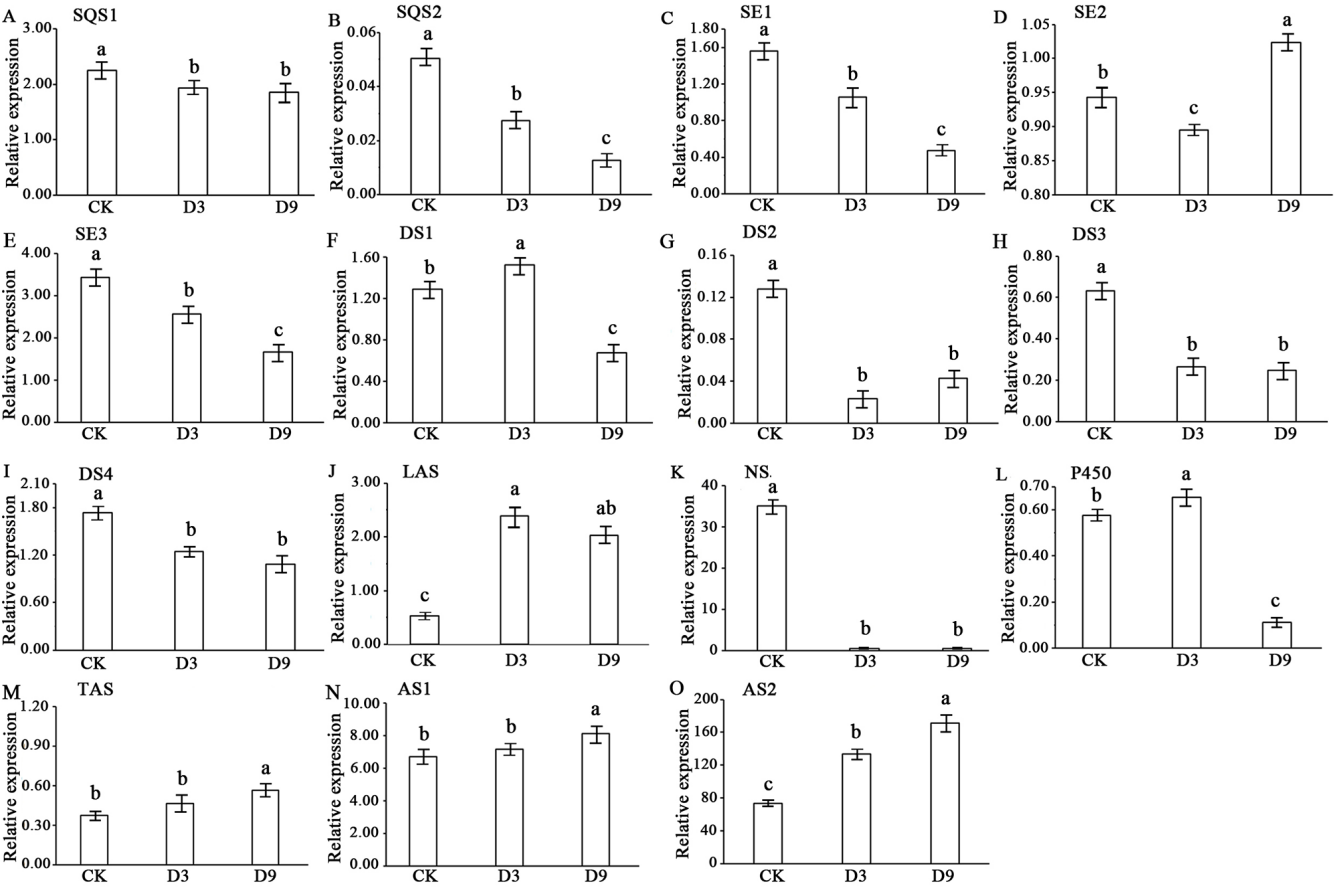
qRT‒PCR analysis of 15 DEGs related to sesquiterpenoid and triterpenoid biosynthesis in *Atractylodes chinensis*. Values represent the mean ± SE (*n* = 3). Error bars indicate standard errors of the mean. Letters indicate significant differences among control, D3, and D9 based on one-way ANOVA (*p* < 0.05). SQS1: TRINITY_DN2734_c0_g1; SQS2: TRINITY_DN11881_c0_g2; SE1: TRINITY_DN1332_c1_g1; SE2: TRINITY_DN626_c0_g1; SE3: TRINITY_DN7063_c0_g3; DS1: TRINITY_DN29281_c0_g1; DS2: TRINITY_DN3072_c1_g1; DS3: TRINITY_DN10738_c0_g2; DS4: TRINITY_DN833_c0_g1; LAS: TRINITY_DN81522_c0_g1; NS: TRINITY_DN3403_c0_g2; P450: TRINITY_DN1163_c1_g1; TAS: TRINITY_DN5420_c0_g1; AS1: TRINITY_DN4574_c0_g1; AS2: TRINITY_DN4574_c0_g3

Using qRT‒PCR analysis, the expression trends of the 15 screened DEGs involved in sesquiterpenoid and triterpenoid biosynthetic pathways were largely consistent with the transcriptome. However, the relative expression levels were different. In D3 and D9, the relative expression levels of *SQS1*, *SQS2*, *SE1*, *SE3*, *DS2*, *DS3*, *DS4*, and *NS* were significantly lower than in the control (Fig. 9A, B, C, E, G, H and I, and 9K). In D9, the expression of *SE2*, *TAS*, *AS1*, and *AS2* were significantly higher than in the control and D3 (Fig. 9D, M and N, and 9O). For *DS1*, *P450*, and *LAS*, the relative expression levels were highest in D3 (Fig. 9F and J, and 9L). The abovementioned results indicate that the transcriptome-based DEG analysis was reliable for identifying drought-responsive genes (Fig. 9).

## Discussion

### Physiological responses in *A. chinensis* are affected by drought stress

RWC is an important indicator of plant water status, which is essential for the normal growth and physiological functions of plants. Maintenance of a higher RWC during drought stress is associated with drought tolerance [30]. In the present study, no significant difference was observed in plant RWC of the drought stress groups compared with that of the control. Plants maintain physiological balance through higher RWC, which is an indicator of drought resistance [31, 32]. To maintain the balance of physiological metabolism under drought stress, plants accumulate small molecular organic compounds, such as proline, soluble sugar, and crude protein. The accumulation of proline is reported as one of the adaptation mechanisms for plants in response to drought stress [33, 34]. In addition to small molecular organic compounds, they are accompanied by changes in antioxidant enzyme activities. Under stress conditions, enhanced antioxidant enzyme activities indicated that the plant exhibited stronger drought resistance [27], which has been reported in previous studies [35–37]. In *Helianthus annuus*, proline and glycine betaine contents and antioxidant enzyme (SOD, POD, and CAT) activities increased when plants were subjected to drought stress [38]. For *A. lancea*, the activities of four antioxidative enzymes (SOD, POD, CAT, and APX) were higher under drought stress than in the control group during the early stage of drought stress [15]. Our observations suggest a similar mechanism in *A. chinensis* affected by drought stress. All seven physiological indices analyzed in this study were significantly higher in D9 than in the control. This may imply that the induction of physiological changes is an adaptation strategy that *A. chinensis* uses to overcome drought stress.

### Transcriptomic responses in *A. chinensis* are affected by drought stress

The change in antioxidative enzyme activities under drought stress is closely related to gene expression [10, 15]. In the present study, we integrated physiological indices and transcriptomic sequencing data to reveal the molecular mechanism of *A. chinensis* seedlings exposed to drought stress. From the transcriptomic data, the total number of DEGs was more than 2.8 times higher in control vs. D9 than in control vs. D3. The number of upregulated DEGs in control vs. D3 was nearly three times that in control vs. D9, and the number of downregulated DEGs in control vs. D9 was more than seven times that in control vs. D3. Consistent with previous studies, severe stress resulted in more downregulated unigenes than early stages of drought stress [28]. Under severe drought stress, more than twofold the quantity of DEGs was identified in *Pugionium cornutum* (L.) *Gaertn* [23].

At D3, more translation, peptide and amide biosynthetic processes were enriched based on GO enrichment analysis in *A. chinensis*. With the prolongation of drought stress, DNA repair-related processes were enriched, indicating higher damage at the DNA level in *A. chinensis*. In addition, physiological, photosynthetic, and secondary metabolite biosynthesis-related categories were enriched, indicating comprehensive defense against drought stress in *A. chinensis*. More GO terms and KEGG pathways were enriched in D9 than in D3. Of the GO terms, three indicated enriched categories related to sesquiterpenoid and triterpenoid biosynthesis in D3, in contrast to five categories enriched in D9. Only one secondary metabolite pathway was enriched in D3, in comparison seven secondary metabolite pathways were enriched in D9, including diterpenoid, phenylpropanoid, sesquiterpenoid and triterpenoid, carotenoid, biotin, steroid, and monoterpenoid biosynthesis. Differences in the expression of unigenes involved in secondary metabolite biosynthesis are closely related to their adaptation to environmental conditions [16]. This may be explained by the fact that drought stress influences the physiological processes of plant cells, triggered by signal transduction processes to accommodate the stimulus from changing concentrations of primary and secondary metabolites that enable the regulation of cell osmotic pressure [39–41]. In plants, drought stress is conducive to the accumulation of secondary metabolites, indicating the high expression of genes and metabolic pathways for their biosynthesis [42].

### The sesquiterpenoid biosynthetic pathway in *A. chinensis* is affected by drought stress

Higher production of secondary metabolites is part of the chemical defense response system associated with increased resistance to environmental stress [43]. This means that secondary metabolites can perform specific stress response functions in plants [30, 44]. For medicinal plants, many functional genes related to the biosynthesis of secondary metabolites have been predicted and screened through transcriptome data analysis [17, 21, 45]. In this study, the biosynthetic pathway of the main bioactive sesquiterpene compounds in *A. chinensis* and its related candidate genes were screened by transcriptome sequencing. The main biosynthesis routes for the terpenoid backbone in plants are derived from either the MVA pathway active in the cytosol or the MEP pathway in the plastids [46–49]. We identified six catalytic enzymes in the MVA pathway, four in the MEP pathway, and three in the FDP regulatory pathway in response to drought stress from the *A. chinensis* transcriptomic data. Using DEG analysis, we detected five enzymes encoding DEGs in the MVA pathway, only one in the MEP pathway, and three in the FDP regulatory pathway. Our previous study revealed that there were six enzymes encoding genes in the MEP pathway screened in the transcriptome of *A. chinensis* of different ages [29]. These results suggest that the MVA pathway is the main biosynthetic pathway of the terpenoid backbone in *A. chinensis* in response to drought stress, which is consistent with previous reports that the MVA pathway supplies precursors of sesquiterpene compounds [46, 50–53].

The main products of the MVA pathway are terpene compounds [54, 55]. The starting enzyme, AACT, catalyzes the entry reaction of the MVA pathway to produce acetoacetyl-CoA [56], which is consequently converted into 3-hydroxy-3-methylglutaryl-CoA by 3-hydroxy-3-methylglutaryl coenzyme A synthase (HMGS) [57]. These two enzyme encoding genes, *AACT* and *HMGS*, were upregulated in D9 in the present study. The HMGR enzyme, a rate-limiting enzyme in the MVA pathway, is an important regulatory site in the terpene biosynthetic pathway [58–62]. Our results revealed that one of the two HMGR-encoding genes were upregulated in D3 and then downregulated in D9. The same expression patterns were found in MK- and MDD-related genes and genes in the FDP regulatory pathway. In *Lactococcus lactis*, the production level of the sesquiterpenoid compound β-sesquiphellandrene increased by 1.25- to 1.60-fold when the HMGR-encoding gene was overexpressed [63]. These upregulated genes play important roles in the biosynthesis of terpenoid backbones.

Isopentenyl-diphosphate (IPP) is reversibly converted into dimethylallyl diphosphate (DMAPP) by isopentenyl-diphosphate delta-isomerase (IDI), indicating the completion of the MVA pathway. The condensation of DMAPP and IPP by farnesyl diphosphate synthase (FPPS) produces FPP, which is a precursor of all terpene compounds [64]. In this study, FPPS and geranylgeranyl pyrophosphate synthase (GGPS) showed similar expression patterns in *A. chinensis*. The highest expression level of FPPS appeared in D3 and then decreased in D9. Two of the three GGPS showed the same patterns, and the other GGPS displayed a gradual increase with prolonged drought stress. These data suggest that moderate drought stress stimulates the higher expression level of enzymes encoding genes in the secondary metabolism pathway, resulting in an increased concentration of secondary metabolites [65, 66]. Previous studies have shown that secondary metabolites can increase under moderate drought conditions but decrease under severe drought stress [67, 68].

Previous studies have shown that drought stress significantly affects the sesquiterpenoid and triterpenoid biosynthetic pathways in *A. lancea* [16] and changes the expression levels of some putative genes related to essential oil biosynthesis in *Mentha piperita* [22]. Our results showed that drought stress changes the expression levels of some putative genes. These genes related to terpenoid backbone biosynthesis and sesquiterpenoid and triterpenoid biosynthesis are the most sensitive to drought stress in the metabolism of terpenoids and polyketides. We further investigated how putative genes in the sesquiterpene compound biosynthetic pathway were affected by drought stress. In this study, drought stress strongly affected the biosynthesis of the sesquiterpenoid and triterpenoid pathways, as evidenced by the up- and downregulation of putative enzyme-encoding genes in the drought-affected *A. chinensis* transcriptome and qRT‒PCR analysis. SQS and SE are rate-limiting enzymes in the triterpenoid biosynthetic pathway [69–71]. The DS enzyme is the first dedicated enzyme for triterpenoid compound biosynthesis [72]. The results of this study suggest that the conversion of FPP into triterpenoids may be reduced due to the downregulation of SQS, SE, and most DS-encoding genes in D3. Interestingly, several downstream triterpenoid synthase enzymes including AS, were upregulated under drought stress. TAS and LAS encoding genes were upregulated in D3 and then slightly downregulated in D9. Upregulated genes play an important role in the biosynthesis of secondary metabolites under drought stress. Genes in secondary metabolite biosynthetic pathways can be upregulated by abiotic stress, leading to secondary metabolite accumulation [30].

Only two sesquiterpene-encoding genes, NS and CYP450, were significantly differentially expressed under drought stress in the *A. chinensis* transcriptome. *NS* was downregulated under drought stress, and *CYP450* was upregulated in D3 and then downregulated in D9. This is consistent with previous studies showing that 10 significantly downregulated genes encoding sesquiterpene synthase were mainly expressed in *A. lancea* [15]. FPP is catalyzed by NS to produce the acyclic sesquiterpene compound (3 S,6E)-nerolidol [73] and catalyzed by CYP450 to generate the germacren sesquiterpene compound solavetivone [74]. Thus it could be stated that drought stress stimulates those genes involved in the triterpenoid biosynthetic pathway to upregulate expression while changing the regulation patterns of putative sesquiterpenoid enzyme-encoding genes.

## Conclusion

In general, our results showed that both genes and enzymatic activity were modified by drought stress in *A. chinensis*. We integrated physiological indices and transcriptome data that display genetic information in *A. chinensis* under drought stress and identified the molecular function and expression patterns of putative unigenes involved in sesquiterpenoid and triterpenoid biosynthetic pathways. This study provides insight into the relationships between water deficits and sesquiterpenoid and triterpenoid biosynthesis in *A. chinensis*.

### Electronic supplementary material

Below is the link to the electronic supplementary material.

Supplementary Material 1

Supplementary Material 2

Supplementary Material 3

Supplementary Material 4

Supplementary Material 5

Supplementary Material 6

## Data Availability

The datasets analyzed during the current study are available at NCBI (SAMN33016714) Sequence Read Archive (SRA): PRJNA930596 (https://dataview.ncbi.nlm.nih.gov/object/PRJNA930596?reviewer=strmegng5mpqeqdt167hhn6i5g) repository.

## Abbreviations

*A. chinensis*: *Atractylodes chinensis* (DC.) Koids
DEGs: Differentially expressed genes
control vs D3: Drought stress for 3 days compared with the control
control vs D9: Drought stress for 9 days compared with the control
qRT‒PCR: Quantitative real‒time PCR
RWC: Relative water content
MDA: Malondialdehyde
SOD: Superoxide dismutase
POD: Peroxidase
CAT: Catalase
APX: Ascorbate peroxidase
MVA: Mevalonate
MEP: Methylerythritol phosphate
AACT: Acetyl-CoA C-acetyltransferase
HMGS: 3-hydroxy-3-methylglutaryl coenzyme A synthase
HMGR: 3-hydroxy-3-methylglutaryl coenzyme A reductase
MK: Mevalonate kinase
PMK: Phosphomevalonate kinase
GPPS: Geranyl diphosphate synthase
FPPS: Farnesyl diphosphate synthase
IDI: Isopentenyl-diphosphate delta-isomerase
MDD: Mevalonate pyrophosphate decarboxylase
FPPS: Farnesyl diphosphate synthase
DXS: 1-deoxy-*D*-xylulose-5-phosphate synthase
DXR: 1-deoxy-*D*-xylulose-5-phosphate reductoisomerase
CMK: 4-diphosphocytidyl-2-*C*-methyl-*D*-erythritol kinase
MDS: 2-c-methyl-d-erythritol 2,4-cyclodiphosphate synthase
IPP: Isoprenenyl diphosphate
FPP: Farnesyl diphosphate
GDS: Germacrene D synthase
GAS: Germacrene A synthase
QHS: Beta-caryophyllene synthase
AFS: Alpha-farnesene synthase
FLDH: Farnesol dehydrogenase
NS: (3 S,6E)-nerolidol synthase
TPS: Sesquiterpene synthase
SQS: Squalene synthase
SE: Squalene epoxidase
DS: Dammarenediol-II synthase
AS: Beta-amyrin synthase
LUS: Lupeol synthase
GS: Geraniol synthase
CYP450: Cytochrome P450
LAS: Lanosterol synthase
TAS: Taraxerol synthase
