# Supplementary material for: Transcriptomic and physiological analysis of atractylodes chinensis in response to drought stress reveals the putative genes related to sesquiterpenoid biosynthesis
Source: BMC Plant Biol. 2024 Feb 6;24:91. doi: 10.1186/s12870-024-04780-8 (PMC10845750; doi:10.1186/s12870-024-04780-8)
Supplement: Supplementary file 1 — Supplementary Material 1 [file 12870_2024_4780_MOESM1_ESM.docx]

**Table S1 Primers of the screened 15 DEGs involved in sesquiterpenoid and triterpenoid biosynthetic pathway**

| No. | Enzymes name | Transcripts ID | Forward primer (5’ to 3’) | Reverse primer (5’ to 3’) |
| --- | --- | --- | --- | --- |
| 1 | SQS1 | TRINITY_DN2734_c0_g1 | GCCTGAATGATATGGTGACAAACGC | TGAAGATTGCCGGATCACGTAAGTC |
| 2 | SQS2 | TRINITY_DN11881_c0_g2 | CTTCGAGCCCTTGACACTGTTGAG | TGCGAAATGCCAGTCAGGATCATAG |
| 3 | SE1 | TRINITY_DN1332_c1_g1 | GCGGAAAGGAGCAAGCCAGATG | ACACCAGCACCAACGATGATAACG |
| 4 | SE2 | TRINITY_DN626_c0_g1 | CGTCTTCTGCTACCTCTACCCTCTG | TTTGCCTCACTCCTTCCGCTTTG |
| 5 | SE3 | TRINITY_DN7063_c0_g3 | AGTTTTGGCAGTCAAGAGTGGGATG | TCTTCAGGGTAGGGCCAATCTCG |
| 6 | DS1 | TRINITY_DN29281_c0_g1 | CCGACGGTTCATGGTATGGCTATTG | TCCAGCAGCATCTAAGCCTCCTAG |
| 7 | DS2 | TRINITY_DN3072_c1_g1 | CAAGATCATGGTTGGGTCGTCTCAG | GGTACAGGAGGACATTCACAGCATC |
| 8 | DS3 | TRINITY_DN10738_c0_g2 | GAGGGACATAGCACTGCATGATGG | CCACTTCCTTCCTCGGCATACAAC |
| 9 | DS4 | TRINITY_DN833_c0_g1 | AGAGCGTGACGAAGTCGAGAGG | TGAGCAAGTCCCCGCAACAATG |
| 10 | LAS | TRINITY_DN81522_c0_g1 | CAATCGCTAGAGCCCGCAAGTG | AGCCCTCCCATTCATAGACTCCAAG |
| 11 | NS | TRINITY_DN30423_c0_g1 | AACAAATCCCAACCTCTCGACACAG | GTTAGTTCGTCCACAGTGCCGTAG |
| 12 | P450 | TRINITY_DN1163_c1_g1 | TAGAAGAAGAGACGGAGAGGCACAG | GATGTATCAGCCCCACCACCAAAG |
| 13 | TAS | TRINITY_DN5420_c0_g1 | GACCTGACGACAACGCCTGTTC | TGCTTCCCGACCACTCATATAGACC |
| 14 | AS1 | TRINITY_DN4574_c0_g1 | TCGTTGCTCCTATCACTCCTCTGG | ACACAAATGTCGTATGCTCCTCCAG |
| 15 | AS2 | TRINITY_DN4574_c0_g3 | GTTGGGAGGCAAATATGGGAGTTTG | TCACTGCTGGGCTTAACTTCTTGC |
